# Microbes in the neonatal intensive care unit resemble those found in the gut of premature infants

**DOI:** 10.1186/2049-2618-2-1

**Published:** 2014-01-28

**Authors:** Brandon Brooks, Brian A Firek, Christopher S Miller, Itai Sharon, Brian C Thomas, Robyn Baker, Michael J Morowitz, Jillian F Banfield

**Affiliations:** 1 Department of Earth and Planetary Sciences, University of California Berkeley, Berkeley, CA 94720, USA; 2 University of Pittsburgh School of Medicine, Pittsburgh, PA 15261, USA; 3 Department of Integrative Biology, University of Colorado Denver, Denver, CO 80202, USA; 4 Division of Newborn Medicine, Children’s Hospital of Pittsburgh of UPMC, Pittsburgh, PA 15224, USA

## Abstract

**Background:**

The source inoculum of gastrointestinal tract (GIT) microbes is largely influenced by delivery mode in full-term infants, but these influences may be decoupled in very low birth weight (VLBW, <1,500 g) neonates via conventional broad-spectrum antibiotic treatment. We hypothesize the built environment (BE), specifically room surfaces frequently touched by humans, is a predominant source of colonizing microbes in the gut of premature VLBW infants. Here, we present the first matched fecal-BE time series analysis of two preterm VLBW neonates housed in a neonatal intensive care unit (NICU) over the first month of life.

**Results:**

Fresh fecal samples were collected every 3 days and metagenomes sequenced on an Illumina HiSeq2000 device. For each fecal sample, approximately 33 swabs were collected from each NICU room from 6 specified areas: sink, feeding and intubation tubing, hands of healthcare providers and parents, general surfaces, and nurse station electronics (keyboard, mouse, and cell phone). Swabs were processed using a recently developed ‘expectation maximization iterative reconstruction of genes from the environment’ (EMIRGE) amplicon pipeline in which full-length 16S rRNA amplicons were sheared and sequenced using an Illumina platform, and short reads reassembled into full-length genes. Over 24,000 full-length 16S rRNA sequences were produced, generating an average of approximately 12,000 operational taxonomic units (OTUs) (clustered at 97% nucleotide identity) per room-infant pair. Dominant gut taxa, including *Staphylococcus epidermidis*, *Klebsiella pneumoniae*, *Bacteroides fragilis*, and *Escherichia coli*, were widely distributed throughout the room environment with many gut colonizers detected in more than half of samples. Reconstructed genomes from infant gut colonizers revealed a suite of genes that confer resistance to antibiotics (for example, tetracycline, fluoroquinolone, and aminoglycoside) and sterilizing agents, which likely offer a competitive advantage in the NICU environment.

**Conclusions:**

We have developed a high-throughput culture-independent approach that integrates room surveys based on full-length 16S rRNA gene sequences with metagenomic analysis of fecal samples collected from infants in the room. The approach enabled identification of discrete ICU reservoirs of microbes that also colonized the infant gut and provided evidence for the presence of certain organisms in the room prior to their detection in the gut.

## Background

From birth to death, humans spend approximately 90% of their time indoors [1]. This realization, coupled with advancements in DNA sequencing technologies, has spawned a new interest in studying buildings as ecosystems. Pioneering efforts have revealed a built environment (BE), a term used here to collectively describe both the biotic and abiotic features of a building structure, that is far more complex than originally imagined [2,3]. Diverse microbial communities have been uncovered in a variety of BEs [4] and surprisingly, from sites engineered to be sterile or near sterile, such as NASA clean rooms [5,6] and high-risk hospital wards [7-10]. Additionally, recent studies characterizing different building types have revealed general trends suggesting a room’s function or architecture dictates the BE’s microbiome [8,11]. Intrabuilding experiments in hospitals have corroborated this notion, showing general use areas, such as waiting rooms and lobbies, have a markedly different microbial community compared to more restrictive hospital zones such as intensive care units [8]. The exchange between the BE microbiome and the human microbiome communities remains unclear; however, the observation that human pathogens are enriched for in hospital settings is of obvious concern [11]. Here, we aimed to characterize the interaction between the BE’s microbiome and the human microbiome through study of very low birth weight (VLBW, <1,500 g) infants housed in a neonatal intensive care unit (NICU) as our model system.

Infants housed in a NICU are well suited to studies that aim to characterize interactions between the BE and occupants. *In utero*, infants are canonically thought to exist in a sterile or near-sterile environment [12]. Acquisition of the microbiome starts at birth and is strongly influenced by mode of delivery [13]. Patterns of colonization in full-term infants tend to follow a well documented trajectory affected by diet, host genotype, and a limited set of other variables, with the infant gut converging on an adult-like state around 2.5 years of life [14,15]. In VLBW infants, early gut succession is characterized by extremely limited diversity, chaotic flux in community composition, and an abundance of opportunistic pathogens [16-19]. It is possible that a high rate of caesarean deliveries and the routine use of broad-spectrum antibiotics during the first week of life serve to decouple VLBW infants from source inoculum introduced during the birthing process. These influences likely render premature infant microbiomes especially susceptible to environmental influences.

There is strong evidence suggesting that the ICU serves as a reservoir of clinically relevant pathogens. ‘Outbreaks’ of disease in ICUs are relatively common, and a recent study estimated at least 38% of all ICU outbreaks could be attributed to microbial sources within the ICU environment, such as equipment, or personnel [20]. In addition, upward of 63% of extremely preterm infants develop life-threatening infections [21]. Epidemiologic investigations indicate environmental sources of infective agents in air [22], infant incubators [23,24], sink drains [25], soap dispensers [26], thermometers [27], and baby toys [28]. Clearly there is a growing need for comprehensive ecological surveys of the hospital BE to better understand the overall process of microbe migration and establishment on and in the body of occupants. Here, we performed the first matched time series characterization of the NICU and infant gut. Our analysis used metagenomic sequencing of microbial community DNA extracted from fecal samples to evaluate the metabolic potential of gut colonizing microorganisms and a recently developed ‘expectation maximization iterative reconstruction of genes from the environment’ (EMIRGE) amplicon protocol to profile the microbial community composition of BE samples collected from six environment types [29]. Our protocol was aimed at addressing the hypothesis that the BE, specifically room surfaces frequently touched by humans, is a predominant source of colonizing microbes in the GI tract of premature infants.

## Methods

### Sample collection

Fecal samples were collected every third day, starting on the third day of life, for 1 month from two infants. Infants were enrolled in the study based on the criteria that they were <31 weeks’ gestation, <1,250 g at birth, and were housed in the same physical location within the NICU during the first month of life. A summary of health-related metadata including antibiotics exposure is provided in Table 1. Fecal samples were collected using a previously established perineal stimulation procedure and were stored at -80°C within 10 minutes [16]. All samples were collected after signed guardian consent was obtained, as outlined in our protocol to the ethical research board of the University of Pittsburgh (IRB PRO11060238). This consent included sample collection permissions and consent to publish study findings.

**Table 1:** Health profile of premature infant cohort

**Characteristic**	**Infant 1**	**Infant 2**
Gestational age	26 3/7 weeks	28 2/7 weeks
Weight	951 g	1,148 g
Multiple gestation	No	Twin
Delivery mode	Vaginal	Vaginal
Chorioamnionitis	Yes	Yes
Day of life (DOL) 1 to 7 antibiotics	Ampicillin, gentamycin	Ampicillin, gentamycin
Other antibiotics	No	DOL 14 to 16, vancomycin, cefotaxime
Feeding initiated	DOL 3, maternal milk	DOL 8, artificial formula
Survive to discharge	Yes	Yes

All samples were obtained from a private-style NICU at Magee-Womens Hospital of the University of Pittsburgh Medical Center. Room samples were collected concurrently with fecal samples and spanned four timepoints on days of collection (9:00, 12:00, 13:00, and 16:00). Most frequently touched surfaces were determined by visual observation and health care provider interviews in the weeks leading up to sample collection. Microbial cells were removed from surfaces using foam tipped swabs (BBL CultureSwab EZ Collection and Transport System, Franklin Lakes, NJ, USA) and a sampling buffer of 0.15 M NaCl and 0.1% Tween20. Six frequently touched areas were processed per infant room: sink, feeding and intubation tubing, hands of healthcare providers and parents, general surfaces, access knobs on the incubator, and nurse station electronics (keyboard, mouse, and cell phone). All samples were placed in a sterile transport tube and stored within 30 minutes at -80°C until further processing.

### DNA extraction and PCR amplification

Frozen fecal samples were thawed on ice and 0.25 g of thawed sample added to tubes with prewarmed (65°C) lysis solution from the PowerSoil DNA Isolation Kit (MoBio Laboratories, Carlsbad, CA, USA). The incubation was conducted for 5 minutes and the manufacturer’s protocol followed thereafter. Swab heads followed the same procedure, except heads were cut with sterilized scissors into the extraction tube before starting the protocol.

DNA extracted from swabs was pooled such that the four timepoints sampled in 1 day per environment were consolidated into one sample. Pooled DNA was used as template for amplification of the full-length 16S rRNA gene with 27 F (5’-AGAGTTTGATCCTGGCTCAG-3’) and 1492R (5’-GGTTACCTTGTTACGACTT-3’) primers [30]. To limit PCR bias, gradient PCR was performed with 5 units/μL of *TaKaRa Ex Taq*™ (Takara Bio Inc., Otsu, Japan) across 7 different annealing temperatures with the following reaction: 1 minute at 94°C; 35 cycles of 1 minute at 94°C, 30 s at 48°C to 58°C (7°C temperature gradient) and 1 minute at 72°C; and a final extension for 7 minutes at 72°C. Amplicons were combined across gradients and cleaned with the QIAquick PCR Purification Kit (Qiagen, Hilden, Germany) as directed by the manufacturer. Cleaned amplicons were quantified via Qubit (Life Technologies, Carlsbad, CA, USA) and input into an Illumina library preparation pipeline.

### Sequencing preparation and sequencing

Illumina library construction followed standard protocols at the University of California Davis DNA Technologies Core Facility (http://dnatech.genomecenter.ucdavis.edu) as previously described [29]. Briefly, amplicons were fragmented to an average size of 225 bp using the Bioruptor NGS (Diagenode, Seraing, Belgium), and sheared fragments were used in a robotic library preparation protocol using the Appollo 324 robot (Integenx, Pleasanton, CA, USA) following the manufacturer’s instructions. Each sample was tagged with unique barcodes consisting of six nucleotides internal to the adapter read as a separate indexing read, and ligated to each fragment. There were 12 cycles of PCR enriched for adapter-ligated fragments before library quantification and validation. Fecal samples underwent the same preparation with two exceptions: (1) genomic DNA was used and (2) DNA was fragmented to 550 bp. Libraries were added, in equimolar amounts, to the Illumina HiSeq 2000 platform. Paired-end sequences were obtained with 100 cycles and the data processed with Casava version 1.8.2. Raw read data has been deposited in the NCBI Short Read Archive (accession number SRP033353).

### EMIRGE assembly of full-length 16S rRNA gene amplicons

EMIRGE is an iterative template-guided assembler that relies on a database of 16S rRNA gene sequences to probabilistically generate full-length 16S rRNA gene sequences and provide the relative abundance of these sequences in the assayed consortia [31]. For the reference database, we used version 108 of the SILVA SSU database, filtered to exclude sequences <1,200 bp and <1,900 bp [32]. To remove closely related sequences, we clustered the database at 97% identity with USEARCH [33]. A total of 1 million paired-end reads from each barcoded library were sampled randomly without replacement to accommodate computational restrictions associated with use of the full dataset. Reads from the subsample from each library were stringently trimmed using Sickle [34] for quality scores <30 and length <60 bp. Trimmed reads were input into an amplicon-optimized version of EMIRGE [29] for assembly using default parameters. A total of 80 iterations were performed for each subsample. EMIRGE-reconstructed sequences without Ns and with an estimated abundance of 0.01% or greater were kept for analysis. Putative chimeras were removed by using the intersection between two chimera detection programs, DECIPHER [35] and UCHIME v6.0 [36] searched against the 2011 Greengenes database [37]. Finally, reconstructed sequences from a spike-in control experiment (data not shown) were removed for downstream analysis. Sequences used in the analysis are publicly available as a project attachment at http://ggkbase.berkeley.edu/NICU-Micro/.

### Metagenomic EMIRGE assembly of 16S rRNA gene

Metagenomic sequencing of 16 fecal samples on 1 lane of an Illumina HiSeq 2000 produced approximately 350 Mbp of 101 bp paired-end reads. Trimmed reads were input into EMIRGE and default parameters run for 80 iterations using the aforementioned database. After the final iteration, 153,980 reads, spanning all samples, were used in reconstructing fecal 16S rRNA sequences. Downstream filtering and analysis of reconstructed 16S rRNA gene sequences from fecal samples followed that of the room samples.

### Community analysis of room and fecal samples

For community analysis, EMIRGE-reconstructed sequences were input into the standard QIIME 1.5.0 workflow [38]. For presence/absence analyses, representative operational taxonomic units (OTUs) were clustered at the <97% identity level using USEARCH [33] and an OTU table was constructed using QIIME’s pick_otus_through_otu_table.py script. An adjusted OTU table that incorporated EMIRGE generated abundances was constructed using an in-house script [29] and is publicly available as a project attachment at http://ggkbase.berkeley.edu/NICU-Micro/. OTUs were aligned to the Greengenes [39] reference alignment (gg_97_otus_4feb2011.fasta) using the PyNAST aligner [40] and a phylogenetic tree built using FastTree v.2.1.3 [41] with default parameters. Beta diversity was calculated from similar trees using Fast UniFrac scores and visualized with principle coordinates analysis (PCoA) [42]. Taxonomy was assigned to each OTU at the genera and/or species level using the Ribosomal Database Project (RDP) classifier [43] at a confidence interval of 0.8 and trained with the same Greengenes database. OTUs were visualized across room-infant pairs in a spring-weighted, edge-embedded network plot by using QIIME’s make_otu_network.py script [38] with the modified OTU table as input.

### Metagenomic assembly and gene prediction

Assemblies were constructed using idba_ud [44] and an iterative implementation of Velvet [45,46]. For idba_ud assemblies, trimmed reads were assembled using default parameters. For the Velvet assemblies, sequence coverage bins representing major genomes in the dataset were identified by first running the program with permissive parameters in which the k-mer size covered the whole range of observed coverages. We summed the k-mer coverages for all contigs generated by this assembly to define the coverage bins (each of which contains one or more genomes). This provided bin-specific expected coverage, k-mer size, coverage cutoff, and coverage collection threshold parameters for the iterative assembly. After each iteration targeting a specific bin, the bin-specific reads were removed from the dataset.

Time-series-coverage-based emergent self-organizing maps (ESOMs) were used to bin scaffolds generated by metagenomic assembly [47]. Genes were predicted and translated into protein sequences using Prodigal [48]. Functional annotation was added with an in-house pipeline [46]. Genome completeness was determined based on the number of single-copy genes and other conserved genes [49,50] identified in each bin. The relative abundance of each organism in each sample was calculated by mapping reads to unique regions on the assembled genomes. Metagenomic assemblies along with their annotations are publicly available at http://ggkbase.berkeley.edu/NICU-Micro/.

### *Enterococcus faecalis* concatenated ribosomal protein phylogeny

For phylogenetic resolution beyond the 16S rRNA gene, 32 highly conserved, single copy ribosomal proteins were used from infant 1 and 2’s assemblies (RpL10, 13, 14, 16, 17, 18, 19, 2, 20, 21, 22, 24, 27, 29, 3, 30, 4, 5, and RpS10, 11, 12, 13, 15, 16, 17, 18, 19, 20, 5, 6, 7, 8). The same genes from recently sequenced *E. faecalis* genomes, in addition to genes from more distantly related taxa, were obtained from the JGI IMG database. Together, each gene set was aligned using MUSCLE 3.8.31 [51,52] and manually curated to remove ambiguously aligned regions and end gaps [53]. The curated alignments were concatenated to form a 32-gene, 39-taxa, 4,101-position alignment. A maximum likelihood phylogeny for the concatenated alignment was conducted using PhyML under the LG + α + γ model of evolution with 100 bootstrap replicates.

## Results

### Stability of NICU room samples over time and space

**Figure 1:**
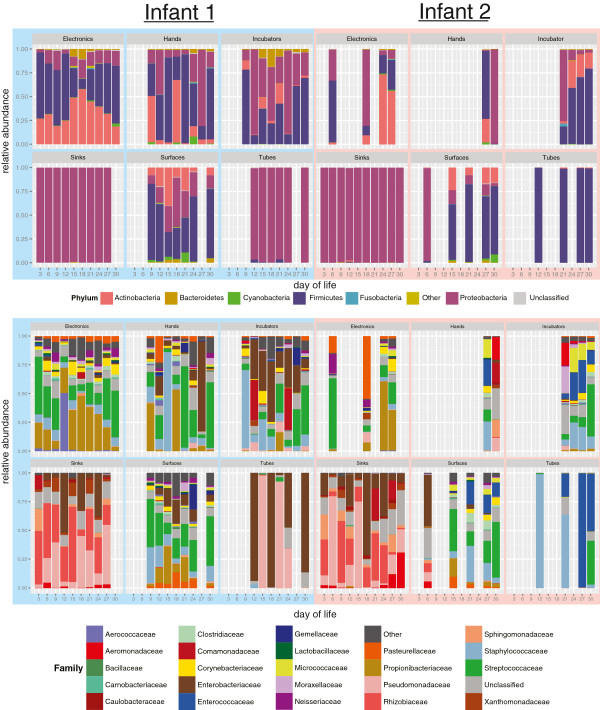
**Taxonomic classification of neonatal intensive care unit (NICU) room microbes for infants 1 and 2.** Phylum-level (top) and family-level (bottom) classifications were assigned using the Ribosomal Database Project (RDP) classifier on assembled full-length 16S rRNA genes. Day of life (DOL) is plotted on the X axis and relative abundance, generated by ‘expectation maximization iterative reconstruction of genes from the environment’ (EMIRGE), is plotted on the Y axis.

**Figure 2:**
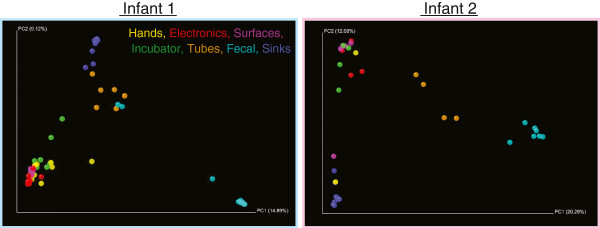
**Principle coordinates analysis (PCoA) based on UniFrac scores of room and gut microbes.** Analysis reveals four discernible ecosystem clusters: skin associated communities, sinks, tubes, and feces.

After sample preparation, 57 and 36 samples amplified successfully and were subsequently analyzed for infant 1 and infant 2, respectively (Table 2). EMIRGE generated approximately 12,000 full-length 16S rRNA sequences and OTUs for each room-infant pair (clustered at the 97% nucleotide identity level). Broadly speaking, species richness decreased from electronics < sinks < surfaces < incubators < hands < tubes, a finding that was corroborated with several alpha diversity indexes (Table 3). Nearly 300 genera were detected in the NICU. To broadly visualize temporal stability of environments across time and space, the phylum level classifications are plotted in Figure 1. Actinobacteria, Firmicutes, and Proteobacteria dominate the sampled environments, with areas most exposed to human skin deposition having the most variation over time. At lower taxonomic levels, similar trends are observed. Based on the 20 most abundant families, frequently touched surfaces are distinct from infrequently touched surfaces (Figure 1). UniFrac distance-based community composition PCoA reveals four discernible ecosystem types (skin associated communities, sinks, tubes, and feces) and confirms clustering of samples prone to skin deposition via touching (Figure 2).

**Table 2:** Sample collection summary and summary of the number of 16S rRNA genes assembled

**Characteristic**	**Infant 1**	**Infant 2**
No. of samples		
Electronics	10	4
Surfaces	7	5
Incubator	8	4
Sink	9	10
Hands	8	2
Tubes	6	4
Fecal	9	7
Total	57	36
No. of EMIRGE sequences		
Electronics	3,359	1,298
Surfaces	2,440	2,205
Incubators	2,270	1,751
Sinks	2,936	4,766
Hands	1,783	812
Tubes	272	198
Fecal	33	32
Total	13,093	11,062
No. of OTUs		
Electronics	3,353	1,293
Surfaces	2,436	2,197
Incubators	2,264	1,749
Sinks	2,933	4,762
Hands	1,781	812
Tubes	271	198
Fecal	33	32
Total	13,071	11,043
Shared OTUs	3,822	
No. of unique OTUs		
Electronics	2,486	1,202
Surfaces	2,211	2,015
Incubators	2,048	1,606
Sinks	2,756	4,453
Hands	1,603	801
Tubes	256	185
Fecal	11	11
Total	10,371	10,273

*EMIRGE* ‘expectation maximization iterative reconstruction of genes from the environment’, *OTU* operational taxonomic unit.

**Table 3:** Alpha diversity indexes from neonatal intensive care unit (NICU) room and fecal samples

**Infant**	**Shannon**		**Simpson**		**Chao 1**	
	**1**	**2**	**1**	**2**	**1**	**2**
Surfaces	8.42848	8.76498	0.997065	0.997677	42,978.9	47,467.2
Electronics	8.36375	8.27527	0.996905	0.996620	45,519.9	33,602.8
Incubators	8.11070	8.76042	0.996291	0.997674	30,216.9	76,196.9
Sinks	8.29052	8.82959	0.996676	0.997687	41,104.6	96,694.1
Hands	7.56186	8.60501	0.993397	0.997322	27,708.1	89,233.5
Tubes	5.06097	5.20681	0.961848	0.963895	1,756.60	1,828.00
Fecal	1.71097	2.10295	0.640741	0.747619	9.70000	13.7000

### Time-series characterization of fecal samples

More than 94% of the reads from infant 1’s samples mapped to scaffolds generated by the idba_ud assembly. Consequently, this assembly was accepted for further analysis. In comparison, the initial idba_ud assembly of metagenomic data from infant 2 was highly fragmented, and less than 40% of reads could be mapped to the assembled scaffolds. Subsequent reassembly of metagenomic data from infant 2’s samples using the iterative Velvet-based assembly approach [54] generated a significantly better result. As <90% of reads could be mapped to the scaffolds generated by the Velvet assembly, this assembly was chosen for further analysis.

**Figure 3:**
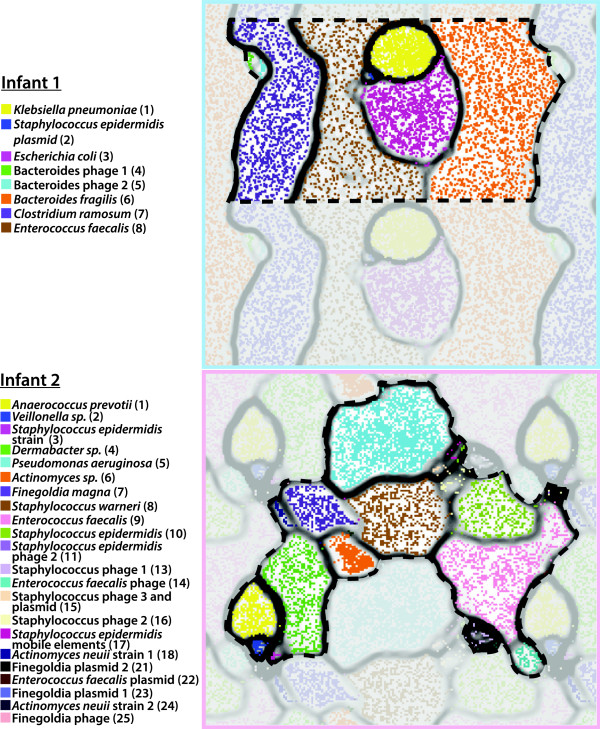
**Time-series coverage emergent self-organizing maps (ESOMs) reveal discrete genome bins for each infant’s dataset.** The underlying ESOMs are shown in a tiled display with each data point colored by its taxonomic assignment. Labels to the left are colored to match their respective data points and numbers in parentheses correspond to the bin numbers in Table 4.

The *de novo* assemblies reconstructed a majority of the genomes for 4 of the 5 and 8 of the 11 most abundant bacterial colonists from infant 1 and infant 2’s metagenomes, respectively. For infant 1, time-series organism abundance patterns in the sample sets analyzed via ESOM (Figure 3) defined five major genome bins for which between 37% and 99% of the single copy genes were identified, based on standard analyses of the single copy gene inventory (Table 4). For infant 2, time-series organism abundance patterns in the sample sets analyzed via ESOM (Figure 3) defined 11 major genome bins for which between 27 and 99% of the single copy genes were identified (Table 4).

**Table 4:** Genome summaries

**Taxa**	**Bin no.**	**bp**	**Contigs**	**N50**	**% GC**	**Cvg**	**% SCG**
Infant 1:							
*Bacteroides fragilis*	6	4,551,095	39	249,654	43.3	1,930.3	99
*Bacteroides* phage1	4	205,842	1	205,842	41.9	2,221.4	0
*Bacteroides* phage2	5	144,903	1	144,903	42.0	2,060.8	0
*Enterococcus faecalis*	8	2,649,897	93	40,945	37.8	7.6	99
*Clostridium ramosum*	7	3,630,043	63	78,436	31.4	23.5	99
*Escherichia coli*	3	5,035,302	53	218,574	50.5	1,254.1	57
*Klebsiella pneumoniae*	1	5,447,442	78	189,741	57.3	345.0	37
*Staphylococcus epidermidis* plasmid	2	20,739	2	11,095	31.5	14.5	0
Infant 2:							
*Actinomyces neuii* strain 1	18	1,580,717	37	280,583	56.9	15.6	27
*Actinomyces neuii* strain 2	24	2,375,188	27	179,095	56.7	17.6	70
*Actinomyces sp.*	6	2,666,449	11	345,356	59.3	55.4	99
*Anaerococcus prevotii*	1	1,599,845	13	225,571	33.1	39.2	99
*Caudovirales* bacteriophage	26	18,308	1	18,308	29.5	1,169.7	0
*Dermabacter sp.*	4	2,040,279	12	289,797	62.8	51.9	90
*Enterococcus faecalis*	9	3,011,019	26	499,183	37.1	147.3	99
*Enterococcus faecalis* phage	14	335,286	39	12,896	34.8	103.7	0
*Enterococcus faecalis* plasmid	22	8,514	2	4,866	30.4	90.6	0
*Finegoldia magna*	7	1,729,913	42	78,482	32.0	93.0	99
*Finegoldia* phage	25	3,168	1	3,168	32.3	138.5	0
*Finegoldia* plasmid 1	23	7,589	2	3,969	33.0	103.4	0
*Finegoldia* plasmid 2	21	28,958	3	15,674	55.4	10.9	0
*Pseudomonas aeruginosa*	5	6,755,599	64	212,603	66.0	51.5	99
*Staphylococcus epidermidis*	10	1,902,759	82	40,484	33.0	65.4	7
*Staphylococcus epidermidis* mobile	17	55,503	10	6,452	31.7	54.5	43
*Staphylococcus epidermidis* phage 2	11	19,082	2	12,983	29.4	84.3	0
*Staphylococcus epidermidis* strain	3	81,754	9	14,965	29.4	67.1	0
*Staphylococcus* phage 1	13	216,785	13	8,080	29.5	45.7	0
*Staphylococcus* phage 2	16	198,742	14	20,782	0.3	79.3	0
*Staphylococcus* phage 3 and plasmid	15	137,609	12	19,343	29.3	67.8	0
*Staphylococcus warneri*	8	2,363,750	22	198,467	32.8	33.9	53
*Veillonella sp.*	2	2,281,484	223	12,637	37.8	56.2	70

Infant 1 and infant 2’s gastrointestinal tract (GIT) microbial communities are distinctly different. Infant 1’s colonization pattern echoes the canonical observation in infant GIT succession that facultative anaerobes dominate early phase colonization whereas late stage colonizers are primarily obligate anaerobes [12]. This shift is observed on day of life 12 in infant 1, but is not observed in infant 2, in whom facultative anaerobes were observed throughout the study period. The metagenomic EMIRGE analyses corroborated the binning-based compositional analyses in that no sequences for new taxa were assembled for scaffolds included in the ESOM. Some 16S rRNA genes were identified in the metagenomic assemblies and match EMIRGE generated sequences with approximately 100% identity. The *E. faecalis* sequence from infant 1 was not identified by EMIRGE due to low abundance, but was extracted from the assembly using RNAmmer for the phylogenetic analysis [55].

### Highly connected BE microbes

**Figure 4:**
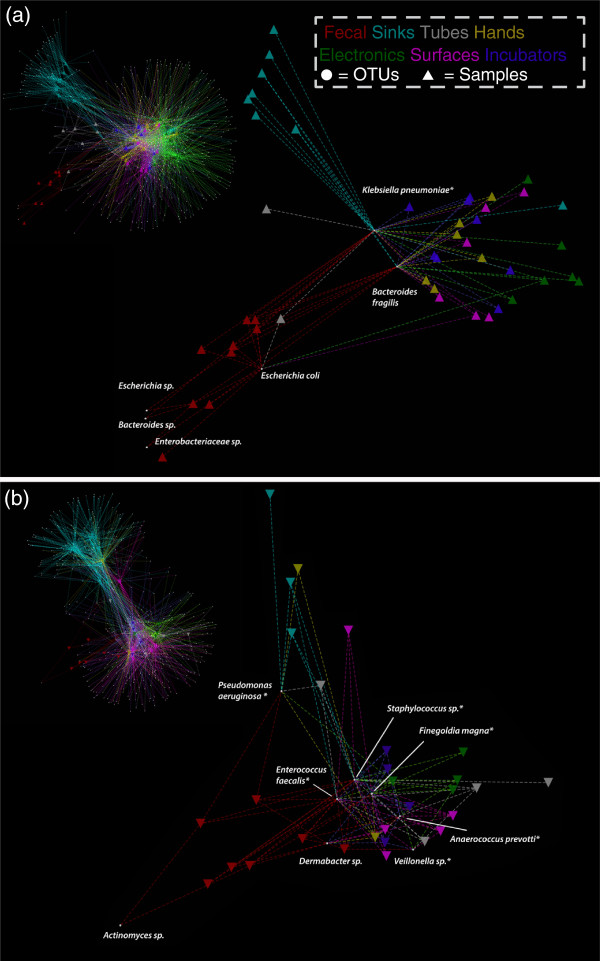
**Spring-weighted edge-embedded network plots of room and fecal operational taxonomic units (OTUs).** Found in two or more samples (infant 1 **(a)**, infant 2 **(b)**). Left, the entire network is displayed. To better visualize the distribution of gut colonizers across room samples, only room samples sharing fecal OTUs are shown in the excerpt (right). Triangles represent samples and circles represent OTUs. The spring weight is derived from ‘expectation maximization iterative reconstruction of genes from the environment’ (EMIRGE) generated abundances and edges are colored by environment type. Each OTU has a taxonomic label and asterisks indicate OTUs detected in room samples before detection in the gut.

The distribution of shared OTUs across sampled sites was visualized through a spring-weighted edge-embedded network plot. To limit the noise from infrequently detected microorganism types, we restricted the plot to OTUs occurring in two or more samples from each infant (Figure 4). The spring weight is derived from EMIRGE generated abundances, and the distribution of OTUs in the plot is governed both by frequency of occurrence and abundance. In Figure 4, the circular white nodes (representing OTUs) found in many environment types (more edges) are pulled closer to the middle of the network whereas OTUs shared by only two samples (fewer edges) are positioned closer to the periphery of the network. The top 5% of most frequently occurring OTUs aggregate in a central cluster in the middle of the network. Similar to the PCoA plot, general clustering is observed based on environment type (that is, skin-associated sites cluster together, as do sink samples). When restricting the network for OTUs only found in fecal samples (Figure 4, enlargements), one can visualize the OTU distribution across the sampled NICU environments. Three highly connected OTUs are present in fecal samples, two of which are in the top 5% most frequently occurring OTUs in infant 1’s room samples. Several of the OTUs in infant 2’s fecal samples fall within the top ten most frequently occurring OTUs in the room environment. Interestingly, infant 2’s most abundant gut colonists, *Staphylococcus sp.* and *E. faecalis*, are the two most frequently occurring OTUs in the room environment.

### The NICU as a reservoir for gut colonists

**Figure 5:**
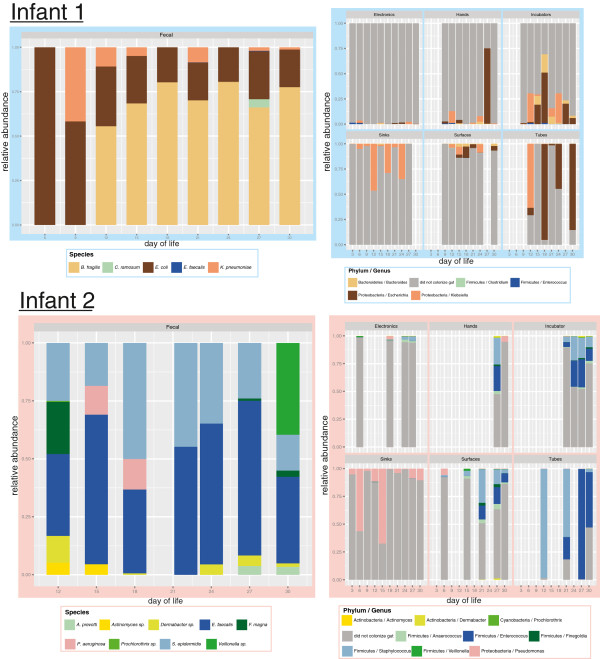
**Community composition of gut colonizing microbes and room microbes through the first month of life.** Time-series characterization of the fecal microbial community (left) and fecal microbes concurrently collected from the room (right) display discrete reservoirs of gut colonizers in the neonatal intensive care unit.

Figure 5 summarizes the gut colonizing organisms found in room samples at the genera level. Typically, for both infants, electronics had the lowest relative abundance of organisms detected in the gut whereas tubing had the highest. Temporal variation of gut genera was extreme in most environments.

**Figure 6:**
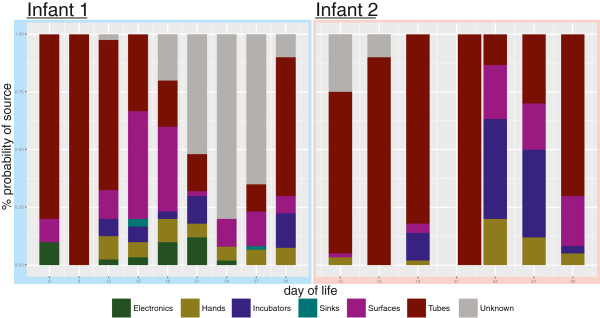
**The most probable source of gut colonizing microbes.** This was generated using the source-sink characterization software, SourceTracker. Neonatal intensive care unit room sequences were designated as putative sources and fecal sequences sinks.

The use of Bayesian microbial source tracking software [56], with the perspective of room samples as the source and fecal samples as the sink, produced mixed results in terms of finding likely gut reservoirs (Figure 6). In infant 1, tubing, surfaces, and electronics had the highest probabilities as sources, but the bloom of *Bacteroides fragilis*, from a source not detected by our sampling regime, lowered the probability of sampled source environments for the latter half of the sampling period. Infant 2’s samples showed the opposite pattern in that early gut colonists migrated from an unknown reservoir, whereas later in sampling, incubator, tubing, surfaces, and hands were the most probable reservoir.

### Shared gut colonizers

**Figure 7:**
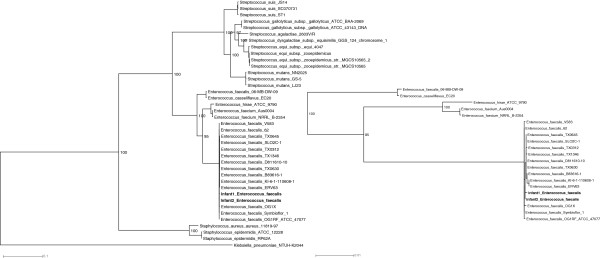
***Enterococcus faecalis *phylogeny using 32 concatenated ribosomal proteins reveals closely related strains.** The maximum likelihood phylogeny of *E. faecalis* strains was based on a concatenation of single-copy, highly conserved ribosomal proteins from our data set and available reference genomes. Bootstrap values greater than 50 are shown. An excerpt of the *E. faecalis* clade is shown to the right.

The infant cohort shared only one gut colonizer, *E. faecalis*, which contained 100% 16S rRNA gene level sequence identity. A higher resolution analysis using a concatenated alignment of 32 highly conserved, single-copy genes show the strains differ by only 2 amino acids across the 4,101 positions. These two *E. faecalis* strains phylogenetically cluster most closely to each other, but are very closely related to other *E. faecalis* strains (Figure 7).

To further explore similarity of shared strains, reads from infant 1 were mapped to infant 2’s assembled contigs. Infant 1’s reads covered 95% of the length of infant 2’s assembly at an average of 4.66X coverage. Read mapping revealed two distinct SNP profiles for infant 1’s reads, a major strain divergent from infant 2’s assembly and a minor strain identical to the strain in infant 2. In all, 77% of the length of infant 2’s *E. faecalis* assembly is covered by infant 1’s reads mapped as mate pairs with no mismatches. This suggests that infant 1’s *E. faecalis* minor strain is the same strain dominating infant 2’s gut. Pheromone-responsive plasmids were found in both infants. The plasmid from infant 2 occurs in low abundance in infant 1 (as expected based on the low representation of *E. faecalis* in infant 1), but with high sequence identity.

### Genes relevant to adaptation to the NICU environment

Analysis of reconstructed genomes for gut microorganisms can lend clues as to how organisms detected in the GIT and room environment are able to persist in the NICU, which is subjected to regular cleaning/sterilization. Numerous antibiotic resistance genes were found in genomes of microorganisms in fecal samples of both infants. A large portion of these were efflux pumps, with representatives from all four families of multidrug transporters: major facilitator superfamily (MFS), small multidrug resistant (SMR), resistance-nodulation-cell division (RND), and multidrug and toxic compound extrusion (MATE) proteins [57]. Particularly interesting are genes encoding the QacA/B MFS, SugE SMR, and MexA/B RND proteins, which are a growing concern in hospitals due to coselection through the practice of combining two or more types of antibiotic treatments [58]. Resistance to multiple types of antibiotics can arise from a single resistance mechanism such as efflux pumping [59]. In addition to antibiotics, these pumps can expel quaternary ammonium compounds (QACs), the active biocide in the detergent used to clean hospital surfaces during the study. Other notable observations were the presence of biofilm forming genes in most colonizers, which can be induced by exposure to aminoglycosides [60], a suite of genes that confer resistance to starvation, and the presence of antibiotic resistance genes encoded on several phage and plasmid genomes, as well as microbial genomes.

## Discussion

Increasing throughput, decreasing cost, and rapid development of informatics and sequencing pipelines has reshaped the field of microbial ecology, allowing researchers to survey a breadth of new environments [34,61-63]. Recently, the first ICU survey to utilize next generation sequencing technology was published [8] and showed a surprising amount of bacterial diversity for an environment under constant attack via aggressive sanitation and antibiotic treatment efforts. The consortia were generally diverse, but some consortia contained a high representation of members of the family Enterobacteriaceae, typically considered to be gut microbes. Shortly after this publication, a study characterizing a snapshot of surfaces and sinks in two NICU rooms corroborated high proportions of fecal coliform bacteria on surface samples [10]. Certainly the NICU has the capacity to retain enteric microbes, but their propensity to migrate to the gut remains unclear.

Next-generation sequencing surveys in the ICU have reported high levels of community diversity. Poza *et al*. found 1,145 distinct OTUs in an ICU in Spain [8] and subsequent studies reported 1,621 and 3,925 OTUs in a NICU in the US and in an Austrian ICU, respectively [9,10]. While comparing these studies is difficult due to differences in sample size and protocols, we can begin to appreciate the need to better understand why so many types of bacteria can be found in a regularly cleaned environment. Our study, the first time series survey of an ICU using next-generation sequencing technologies, unveiled over 20,000 OTUs across 2 NICU rooms occupied by different infants with partial time overlap. Our study is distinct from prior NICU surveys in that it used amplicon-EMIRGE, a 16S rRNA gene assembly software which can be more sensitive in OTU detection [29] and provide increased confidence when making lower taxonomic level classifications [64]. The increase in OTUs from study to study might be attributed to increases in sequencing read lengths and, in this study, increased information from reassembled, full-length genes, but the biological relevance of this increase is unclear. Notably, of the over 20,000 OTUs characterized here, only 984 were found in 2 or more samples. Further surveys are needed, integrating time-series sampling and samples from multiple surface types from different hospitals, to better characterize the expected number of OTUs in an ICU and the implications of this number for ICU occupants.

The increased sensitivity provided by EMIRGE was helpful when evaluating temporal patterns, especially pertaining to source-sink characterization. Similarly, our source-sink analyses benefited from the increased number of samples and timepoints relative to prior studies [8-10], which did not attempt to identify source-sink relationships. The SourceTracker results suggest the most probable room reservoir for gut colonists is tubing followed by surfaces, incubators, and hands (Figure 6). The tubing area sampled, the hub of the silastic nasogastric feeding tube, is the closest in proximity to the infant and, since SourceTracker is not bidirectional, it is difficult to tease out the directionality in this exchange [56]. Incubators from both infants also appear to mirror successional patterns in the infant’s GIT, but without finer scale temporal sampling it is difficult to determine the true source and sink. The observation that hands tend to show a variable amount of potential fecal colonist is likely due to the variability in sampling and hand hygiene, as hand samples were taken both before and after infants received care from healthcare providers. A good example of this is infant 1’s DOL 27 hand sample in which the large spike in *Escherichia* likely came from a swab collected directly after contact with the infant (Figure 5).

Given the large inventory of sequences and the time-series dataset, it was possible to identify likely reservoirs of microorganisms in the room environment, prior to their appearance in the GIT (for example, the asterisked OTUs in Figure 4). Many of these sequences had perfect or near perfect identity between room and GIT 16S rRNA genes. Two notable examples include the *Klebsiella pneumoniae* in infant 1 and *Finegoldia magna* in infant 2, whose fecal to room sequence best hits averaged 99.4% and 99.6% identity respectively. Infant 1’s *K. pneumoniae* is first detectable in the gut on DOL 9, but NICU samples first detect the organism on electronic and sink samples starting at DOL 3, our earliest sampling point. Interestingly, the *K. pneumoniae* is outcompeted in the gut, yet is reintroduced on two separate occasions. This could be a byproduct of our detection limits, but the relatively high abundance of *Klebsiella* in many NICU samples and its availability at all timepoints, suggests the opportunity for reinoculation from multiple room reservoirs. The *F. magna* in infant 2’s samples exhibit similar patterns in that it is initially a high-ranking taxa that is out competed by other Firmicutes, but is reintroduced later in the time series.

If the environment is a reservoir for gut colonizing microbes in our cohort, then it is likely infants housed in close proximity will share the same strain. The 16S rRNA gene survey shows the availability of reservoirs of colonizing populations (likely with multiple strain variants) in the infant’s immediate environment. However, it cannot discriminate at the strain level, so the mere existence of a phylotype in the room prior to gut colonization is not a direct measure of BE to infant transfer. The current work resolves this, by using extensive genome sequence comparison of *E. faecalis* from the gut of two infants housed in the same ward to establish that environment to room occupant transfer occurs in the NICU. The mode of acquisition of infant 2’s abundant strain by infant 1 is unclear, but nosocomial infection by enterococci is not uncommon.

Enterococci are particularly difficult to classify due the plasticity of their genomes. Upwards of 25% of *E. faecalis* genomes may be comprised of mobile or acquired elements [65]. Recent experiments attribute this genome flexibility partially to the ability to produce transconjugant hybrid strains in which several 100 kb fragments can be transferred between donor and recipient strain [66]. Transfer of these genome fragments is dependent on pheromone-responsive plasmids, which were found in all strains studied here. The ability to form hybrids not only confounds the ability to confirm identical strains, unless the entire genome has been recovered, it also provides a competitive advantage in the hospital BE where enterococci have been problematic for decades [65,67]. Enterococci are notoriously hardy and are able to persist on medical equipment and hospital surfaces for long periods of time [65,68]. They are able to withstand chlorine, heat, some alcohol treatments, and possibly most concerning, several types of antibiotics [65]. Their genome plasticity and ability to easily acquire new genes from other strains make them particularly well suited to thrive in the hospital environment.

Gut colonists must withstand selective pressures both inside and out of the gut. Two obvious forms of selection in the NICU come from hospital cleaning and the broad use of antibiotics. All rooms were cleaned daily using wet solutions containing QACs and all infants were administered multiple types of antibiotics. Incorrect administration of biocides, through misuse or unintended mixing with existing fluids (that is, water from sink samples or removing sanitizing agents via water rinsing), could enrich for resistance genes [69]. Even if used to factory standards, if surface-dried cells or biofilms remain, biocide activity could be ineffective and contribute to cross resistance to biocides and antibiotics [70]. Biofilm forming communities can be upwards of 1,000 times more resistant to QACs than their planktonic forms [71] and biofilm formation can be triggered by the types of antibiotics administered in this study [60]. This may be a contributing factor as to why a recent study found enteric microbial communities to be relatively unaltered before and after routine NICU surface cleaning [72]. Certain types of biofilms in many *Enterobacteriaceae*, including those studied here, contain amyloid fibers, called curli. Curli have been implicated in adhesion to abiotic surfaces, such as polystyrene, Teflon, and stainless steel, and contribute to adhesion to host epithelial cells and invasion by *Escherichia coli* in the gut [73]. This type of dual-purpose adaptation may allow enteric organisms to persist on NICU surfaces until transmission to a more favorable environment such as the gut. Efflux pumps are another multipurpose adaptation conferring competitive advantages inside and out of the gut. Numerous pumps from every major class of efflux pump were identified here and, collectively, can function to pump out QACs and administered antibiotics. Previous studies have positively correlated high QAC minimum inhibitory concentrations (MICs) with increased antibiotic resistance markers in enteric microbes [74], indicating biocide efflux may be an important function for microbes in the ICU. Efflux and biofilm formation are two of many possible explanations as to how colonizers combat both biocides administered during NICU cleaning and host-administered antibiotics.

## Conclusions

Through a time series analysis using full-length rRNA gene sequences, we have established that organisms that appear in the GI tract in the early phase of colonization have reservoirs in the room environment. The findings point to a scenario in which gut microbes are introduced from room sources, thrive in the gut, and are disseminated to the immediate environment, creating a cycle of room to infant colonization. The research also highlights the value of extensive genome comparisons to link colonists from different individuals, an approach that in the future may also target populations sampled directly from room reservoirs.

## Abbreviations

BE: Built environment; DOL: Day of life; ESOM: Emergent self-organizing map; GIT: Gastrointestinal tract; ICU: Intensive care unit; MATE: Multidrug and toxic compound extrusion; MFS: Major facilitator superfamily; MIC: Minimum inhibitory concentration; NICU: Neonatal intensive care unit; OTU: Operational taxonomic unit; PCoA: Principle coordinates analysis; QAC: Quaternary ammonium compound; RND: Resistance-nodulation-cell division; SMR: Small multidrug resistant; VLBW: Very low birth weight.

## Competing interests

The authors declare that they have no competing interests.

## Authors’ contributions

JFB, MJM, and BB conceived of the project. RB organized cohort recruitment and sample collections. BAF conducted nucleic acid extractions and BB conducted the amplification reactions. IS and BB conducted the metagenomic assemblies and BCT provided annotations. CSM and BB conducted the 16S rRNA gene reconstructions. BB and JFB wrote the final manuscript. All authors have read and approve the manuscript.
